# Neurodegenerative Disorders with Hair Abnormalities: An Emergency Room Consultation for Dermatologists

**DOI:** 10.4103/0974-7753.51929

**Published:** 2009

**Authors:** Arun C Inamadar, Aparna Palit

**Affiliations:** Departments of Dermatology, Venereology and Leprosy, SBMP Medical College, Hospital and Research Center, BLDE University, Bijapur, Karnataka, India

**Keywords:** Elejalde disease, Menkes kinky hair syndrome, hair abnormalities

## Abstract

Menke′s syndrome and Elejalde disease are the two neurodegenerative disorders of dermatological interest. These patients present with characteristic hair changes which may be of diagnostic value in resource-poor setup where facilities for specific genetic analysis are not available. Simple light microscopic examination of hair may be a helpful diagnostic tool to pick up such cases.

## INTRODUCTION

Neurodegenerative disorders include a group of diseases where progressive, irreversible neuronal damage occurs in areas of central nervous system. Two neurodegenerative disorders of dermatologists′ interest are Elejalde disease and Menkes kinky hair syndrome. Both these disorders present with irreversible brain damage and early death of the affected individual. Though the neurological signs and symptoms of these patients are indistinguishable from other similar disorders, typical hair abnormalities seen in these patients help to differentiate these conditions.

## CASE REPORTS

### Case 1

A two-year-old boy was admitted to the pediatric intensive care unit with history of generalized seizure and loss of consciousness for the past two days. Dermatological consultation was sought for unusual appearance of the patient′s hair. He was the first child of consanguineous parents. The mother′s pregnancy was uneventful and the child was apparently normal at birth, but manifested psychomotor retardation and episodes of seizures starting at late infancy. The two elder siblings of the patients were apparently normal. The child′s growth parameters were lower compared to age and sex-related reference scale.

**Figure 1:**
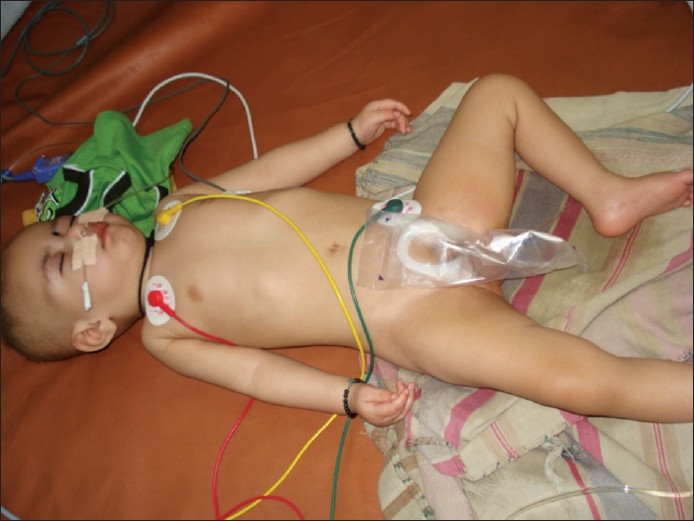
A fair-complexioned baby in unconscious state

**Figure 2:**
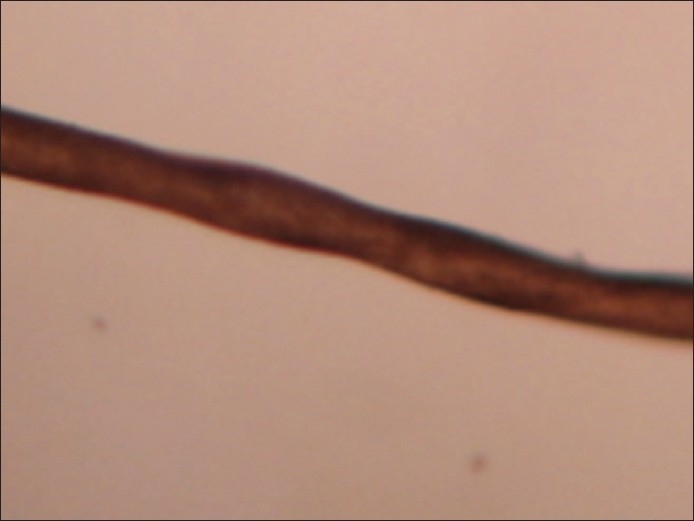
Light microscopy of hair showing pili torti (×–200)

Clinical examination revealed an unconscious, fair-complexioned baby (as compared to parents and other siblings) with flat nasal bridge and pudgy cheeks [Figure 1]. The scalp hairs were sparse, thin, light-brown in color, short and extended out from the scalp giving a steel-wool appearance. Few hairs were plucked for light microscopy which revealed a series of regularly spaced twists along the light-colored hair shafts [Figure 2]. A provisional diagnosis of Menke′s syndrome was made.

Further investigations to establish the diagnosis (serum copper and ceruloplasmin levels) were planned, but the patient succumbed rapidly to death resulting from irreversible brain damage.

### Case 2

A 10-year-old girl was admitted with a history of convulsion followed by unconsciousness since day one. She was admitted to the hospital six months back for a similar episode and diagnostic lumber puncture was done. Though the CSF analysis report was not confirmative of tuberculous meningitis, antitubercular chemotherapy was started on benefit of doubt and continued, which the patient had taken regularly till the day of present admission. The patient was referred to dermatologists for evaluation of her unusual skin and hair color.

**Figure 3:**
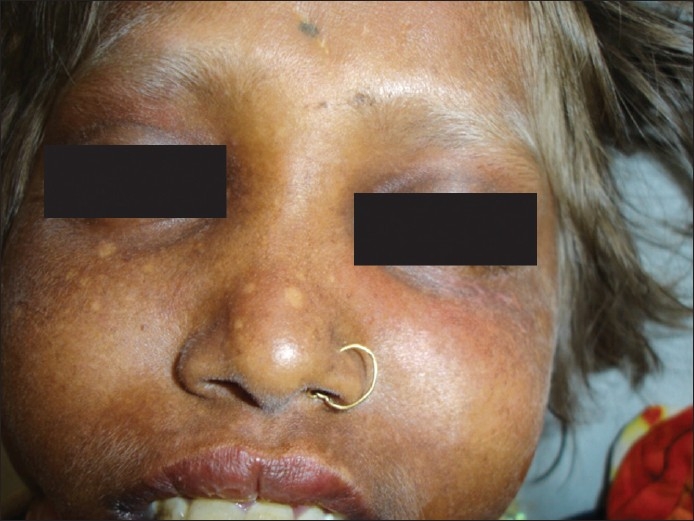
Bronze-tan of photo-exposed skin with silvery shine of the hair

On examination, the patient was deeply comatose with generalized hypotonia; there was no focal neurodeficit. Cutaneous examination revealed a diffuse bronze-tan of her photo-exposed body parts like face, neck, and extremities along with scattered freckles. Hair showed a silvery shine [Figure 3]. CT scan of the brain revealed diffuse cerebral edema. Her parents were consanguineously married. According to them, the patient′s intelligence was normal and she was a fourth standard student. She and her six-year-old younger sister had this type of skin and hair color since early childhood which was progressive and had not been observed in any other family member. The younger sibling did not suffer from any neurological abnormality till that age.

**Figure 4:**
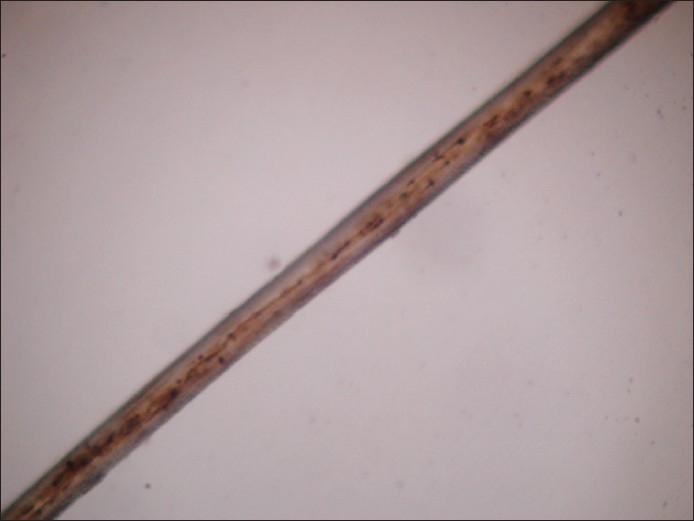
Irregular clumps of melanin along the hair shaft (×–200)

Few hairs were plucked from patient′s scalp and examined under microscope, which revealed irregular clumps of melanin along the hair shaft [Figure 4]. A skin biopsy was done from the forearm; H and E stained histopathological section showed irregular-sized melanin granules dispersed along the basal layer of epidermis. A provisional diagnosis of Elejalde disease was made.

The patient was put on conventional anticerebral edema therapy and regained consciousness within a week without any residual neurodeficit.

## DISCUSSION

Menke′s syndrome is an X-linked recessive neurodegenerative disorder affecting male children.[1] It is a multisystem disease with neurological abnormalities predominating the clinical picture. There is mutation of ATP 7A gene (MNK; chromosome Xq13.3) which encodes a copper transporting enzyme adenosine triphosphatase responsible for intestinal absorption of copper.[2]

Clinical manifestations start by 2-6 months of age as lethargy and psychomotor delay. Temperature instability is common giving rise to frequent episodes of hypothermia. Progressive neurological deterioration punctuated by intractable seizure is common.[1] Scalp hairs are normal at birth but soon become finer, dull, hypopigmented, sparse and brittle standing on end, giving rise to a ′steel-wool′ look and feel.[2] Eyebrow hairs may also be involved.[2] The affected children have a cherubic facies which is typically coarse with depressed nasal bridge and an exaggerated ′cupid′s bow arch of the upper lip′.[2,3] Other features in a full-blown case include soft, doughy skin with a lighter color, joint laxity, elongation, tortuosity and aneurysm of medium-caliber arteries, diverticulosis of urinary bladder, recurrent infections, and osteoporosis resulting in frequent fractures.[1,4] Usual outcome of the disease is death by 3-5 years of age.[1] Mothers and sisters of affected children (obligate female carriers) may show streaks of hypopigmentation or patchy areas of pili torti on scalp.[5] Occurrence of the disease among females has been reported.[6]

Low plasma copper and ceruloplasmin levels are diagnostic of the condition. Radiography of long bones reveals widening of metaphysic with spurring, and is of diagnostic help.[1] Light microscopy of hair shows pili torti in most cases and monilethrix/trichorrhexis nodosa less frequently.[1]

Parenteral copper-histidine administration started during perinatal period may halt the progressive neurodegeneration in some cases.[1] This therapy increases serum copper level and may help in survival till adolescence.[6]

Elejalde disease (neuroectodermal melanolysosomal disease) is a rare, autosomal recessive neurodegenerative disorder.[7] In addition to neurological abnormalities, patients with Elejalde disease show features of pigmentary dilution (bronze-tan and silvery hair) and thus more frequently categorized with other disorders in ′silvery hair syndromes′, i.e. Chediak-Higashi and Griscelli syndrome. Pathomechanism of this disorder is yet unclear but some authorities consider this as an allelic variant of Griscelli′s syndrome with a mutation in MYO5A.[8]

Neurological manifestations may be present at birth or develop thereafter. These include seizure, severe hypotonia, focal neurodeficits like flaccid/spastic paraplegia, quadriplegia, or ataxia.[9] Ocular abnormalities are common manifesting as nystagmus or diplopia. Neurological involvements are severe, progressive, and irreversible, and maximum survival reported in the literature is only up to the early years of second decade.[10]

The affected children have a peculiar bronze-tan on the photo-exposed body parts and a lighter color on covered body parts. There is a silvery shine of the scalp-hair, eyebrows and eyelashes, characteristic of this disorder. Light microscopy of the hair reveals irregular clumping of melanin along the hair shafts, and histopathological examination of the bronze-tanned skin reveals irregular clumping of melanin granules along the basal layer.[7]

Pinpointing the diagnosis of neurodegenerative disorders may be difficult as the common presentation in all is a progressive neurological deterioration. This is more so in resource-poor setup, where advanced investigative tools are not available. Menke′s syndrome and Elejalde disease are two such disorders in which the affected children present with neurological emergencies may end up with a fatal outcome even before the establishment of clinical diagnosis. Dermatological consultation is helpful in such cases to reach a provisional diagnosis and simple light microscopic examination of the hair shaft provides a significant clue to the diagnosis.
